# Activating Sirt1 by resveratrol suppresses Nav1.7 expression in DRG through miR-182 and alleviates neuropathic pain in rats

**DOI:** 10.1080/19336950.2020.1732003

**Published:** 2020-02-22

**Authors:** Qianqian Jia, Wenze Dong, Liwei Zhang, Xiaochun Yang

**Affiliations:** Department of Anesthesiology, First Hospital of Qinhuangdao, Qinhuangdao, Hebei, China

**Keywords:** Neuropathic pain, Sirt1, resveratrol, miR-182, Nav1.7

## Abstract

Neuropathic pain is clinically unsatisfactorily treated because of unclear mechanisms. The present study aims to explore the concrete mechanisms underlying the alleviation of resveratrol-activated silent information regulator 1 (Sirt1) to chronic constriction injury (CCI)-induced neuropathic pain. CCI surgery was conducted to the unilateral sciatic nerve of male Sprague-Dawley rats to induce neuropathic pain experimentally. Resveratrol with or without miR-182 antagomir were administered to CCI rats via intrathecal catheter. Behavioral tests including paw withdrawal threshold (PWT) and paw withdrawal latency (PWL) were conducted to explore mechanical allodynia and thermal hyperalgesia. Western blot, qRT-PCR were used to detect the expression levels of Sirt1, miR-182, and Nav1.7 in CCI dorsal root ganglions (DRGs). CCI rats displayed lower PWT and PWL compared with the sham control. Also, the CCI DRGs displayed lower Sirt1 and miR-182 expression as well as higher Nav1.7 expression, which would be almost reversed by resveratrol treatment for 4 successive days. We also found that miR-182 expression inhibition erased the analgesia effect of resveratrol to CCI–induced neuropathic pain possibly through upregulating Nav1.7 expression. In summary, resveratrol alleviated CCI–induced neuropathic pain, possibly through activating Sirt1 to suppress Nav1.7 expression via upregulating miR-182 expression in CCI DRGs.

## Introduction

Neuropathic pain is featured by allodynia, hyperalgesia, spontaneous and evoked pain after a lesion or disease in central or peripheral nervous system[1]. Neuropathic pain is clinically unsatisfactorily treated as a result of unclear mechanisms. Hence it is necessary to figure out the concrete molecular mechanisms underlying the pathogenesis of neuropathic pain in order to obtain convincing strategies to cure this chronic condition.

Evidences illustrate that epigenetic regulations, such as histone modifications and noncoding RNAs, can serve as promising targets for neuropathic pain treatment [2,3]. Abnormal histone acetylation is reported to be involved in the development of neuropathic pain. Ding HH et al. reported the contribution of histone hyperacetylation in the pathogenesis of ventral root transection-induced neuropathic pain [4]. Silent information regulator 1(Sirt1), a classic III histone deacetylase, has been reported to relieve neuropathic pain via different mechanisms. Sirt1 alleviates neuropathic pain related with diabetic pathology, chemotherapeutic drug, and chronic constriction injury (CCI) [5–8]. Sirt1 is now a promising target for neuropathic pain. While the concrete molecular mechanisms underlying Sirt1-related alleviation of neuropathic pain remains to be well clarified.

Resveratrol (3,4',5-trihydroxystilbene) is a natural polyphenolic compound extracted from some fruits and vegetables, including grapes. Resveratrol possesses multiple biological properties, such as anti-oxidative stress, anti-tumor, as well as anti–inflammatory activities [9–11]. Resveratrol displays significant neuropathic pain relief effect with different mechanisms, including balancing inflammation-related cytokines release [12]. As a natural agonist of Sirt1 resveratrol relieves paclitaxel-induced neuropathic pain via activating Sirt1-related signaling pathway [6]. Yin Q et al. pointed that resveratrol exerts the antinociceptive effects via activating spinal Sirt1 of CCI rats. The downstream effector and pathway after resveratrol-initiated Sirt1 activation hasn’t been well elucidated. Considering miR-182 is the downstream effector of Sirt1 and miR-182 is reported to decrease Nav1.7 expression, one promising effector protein of neuropathic pain [13], to relive symptoms related with neuropathic pain [14], we hypothesized that Sirt1 might downregulated Nav1.7 expression via miR-182.

In the present study, the neuropathic pain rat model was established via CCI surgery to investigate the potential effect of resveratrol on CCI–induced neuropathic pain and the role of Sirt1/miR-182/voltage‐gated sodium channel 1.7 (Nav1.7) axis during the relief of this chronic pain.

## Methods and materials

### Animals

Male Sprague-Dawley rats (200–300 g) were purchased from SLAC (Shanghai, China). Rats were kept under 22°C ± 2°C, 12-h light/dark cycle. All animal experiments were performed under the approval of the Animal Research Ethics Committee of First Hospital of Qinhuangdao following guidelines for National Institutes of health for the Care and Use of Laboratory Animals.

### Neuropathic pain modeling

CCI surgery was performed to induce neuropathic pain experimentally using the procedures previously described [15]. Briefly, rats were anesthetized by intraperitoneal injecting 50 mg/kg pentobarbital and placed in the right-lateral position, then the left sciatic nerve was exposed and loosely tied four ligatures by a 4–0 chromic gut at 1-mm intervals. Animals in the sham group underwent the same procedures without nerve ligation.

### Intrathecal catheter placement

Intrathecal catheter were placed lumbosacrally and verified according to the methods reported previously [6]. Briefly, a 2-cm incision was made for the first step longitudinally along L4-L5 under anesthesia condition. A PE-10 catheter which was filled with sterile heparin saline was then sticked into the subarachnoid space through the 2-cm incision and further inserted about 1 cm caudally to make sure the tip of the catheter was located at the lumbar spinal level of the rat. Finally, 5 μL of 2% lidocaine was intrathecal injected and rats that developed transient paralysis in the hind limbs were considered to be successfully implanted intrathecal catheter. The cannulated rats were placed individually and recovered for 7 days. The rats that exhibited normally in the behavioral test were qualified for further CCI surgery.

### Grouping and drug administration

Resveratrol (#R5010) was purchased from Sigma-Aldrich Co. LLC, St. Louis, Missouri and dissolved in 100% DMSO. Sirt1 inhibitor EX527 (#E7034) and Sirt1 activator SRT1720 (#567,860) were also purchased from Sigma-Aldrich, and dissolved in DMSO. miR-182 antagomir and antagomir negative control were synthesized by BGI-Shenzhen (Shenzhen, China) using the sequences listed previously[14]. Most drugs were administered via the pre-placed intrathecal catheter once per day 7 days after CCI for 4 successive days except EX527, which was injected s.c. with the same time pattern. Rats were randomly divided into the following groups. For the CCI group, rats received CCI surgery to establish neuropathic pain model. For the sham group, rats received the same surgery procedures except nerve ligation. For sham+EX527 group, sham animals were injected 10 mg/kg EX527. For CCI+SRT1720, 5 μl SRT1720 (5 μg) was injected into CCI rats. For the CCI+ Resveratrol, 10 μl resveratrol (300 μg) was injected. For the CCI+Res.+miR-182antagomir group, 10 μl resveratrol (300 μg) as well as 2 μl miR-182 antagomir (20 μM) were injected. For CCI+Res.+miR-182anta. -N.C group, 10 μl resveratrol as well as 2 μl miR-182 antagomir negative control (20 μM) were injected.

### Behavioral tests

Behavioral tests were performed at designed timings, 0, 3, 7, 10, 14 d after CCI surgery for neuropathic pain model verification, and 0, 3, 7, 8, 9, 10, 11 d after CCI for the other experiments. To evaluate mechanical allodynia, paw withdrawal threshold (PWT) of bilateral hind paws in response to mechanical stimulation of the calibrated von Frey filaments (Stoelting, WoodDale, USA), was detected. Rats were placed in plastic partitions individually with a metal mesh grid floor and assigned 20 min to acclimate to the test environment. The interval between successive two stimuli was 5–10 s and bending force ranged from 2 to 26 × g. The 50% PWT was evaluated by the previously described up-down method [16]. Left (ipsilateral to CCI) and right (contralateral to CCI) hind paws were tested. To evaluate thermal hyperalgesia, paw withdrawal latency (PWL) of bilateral hind paws in response to heat was determined. Single rat was placed in a plastic chamber on a glass platform equipped with radiant heater. The time period beginning from the application of radiant heat to the withdrawal of hind paw was defined as PWL. Each hind paw was detected for 4 trials with 10-min interval and the last 3 trials were averaged to get the final PWL value.

### Western blot

The L4-L6 dorsal root ganglions (DRGs) of rats from different groups were sampled at specific timepoints, 0, 3, 7, and 14 d following CCI surgery. After 3 washes with icy PBS, the dissected tissues were rapidly transferred into the RIPA lysis buffer (#20-188, 0.5 M Tris-HCl, pH 7.4, 1.5 M NaCl, 2.5% deoxycholic acid, 10% NP-40, and 10 mM EDTA) containing protease inhibitor (#I3786), all from [17]. Total protein was determined by a BCA kit (#P0012, Beyotime, Shanghai, China). For each sample 30 μg protein was loaded, then separated on 10% SDS-PAGE gel, and transferred to a PVDF membrane (#IPVH00010, Merk Millipore, Billerica, MA). Then the membrane was blocked with 5% bovine serum albumin for 2 h at room temperature and incubated with primary antibodies, rabbit anti-Sirt1 (#ab220807, Abcam, Cambridge, UK), mouse anti-Nav1.7 (#ab85015, 1:500, Abcam), and rabbit anti-GAPDH (#ab9485, 1:2000, Abcam, Cambridge, MA, USA), at 4°C overnights. After 2-h incubation at room temperature with corresponding horseradish peroxidase-conjugated secondary antibodies (#AB6721 and #ab6728, 1:3000, Abcam), the targeted proteins were detected using Pierce ECL Western Blotting reagents (#32,106, ThermoFisher, Rockford, IL, USA) and imaged using FluorChem E (Alphalmager Proteinsimple, San Jose, California). GAPDH served as the inner control.

### Quantitative real-time polymerase chain reaction (qRT-PCR)

Total RNA was extracted immediately from the isolated L4-L6 DRGs of rats from different groups at specific timepoints, 0, 3, 7, and 14 d after CCI injury using the TRizol reagent (Life technologies, Carlsbad, CA, USA) according to the instructions. One microgram RNA for each sample was reversed to synthesize the first-strand cDNA using the cDNA synthesis Kit (TaKaRa, Dalian, China). The expression of Sirt1 mRNA and miR-182 were determined by the SYBR Green Premix Ex Taq (TaKaRa). Real-time PCR was performed on the 7900HT fast real-time PCR system (Applied Biosystems, New York, NY, USA). The primers of Sirt1, miR-182, GAPDH, and U6 were synthesized according to the sequences previously prescribed [14,18]. GAPDH and U6 served as inner control to Sirt1 and miR-182, respectively. The amplification program was set as 1-min pre-incubation at 98°C, followed by 40 cycles as 98°C for 10 s, 56°C for 20 s, and 72°C for 30 s in 1 cycle. The comparative 2^−ΔΔCt^ method was applied to quantify the relative expression of Sirt1 and miR-182 to the inner control GAPDH and U6, respectively.

### Sirt1 activity detection

Sirt1 activity was explored by measuring the levels of acetyl histone H3 (Ac-H3), a substrate of Sirt1, in DRG samples at day 11 after CCI. Ac-H3, negatively related with Sirt1 activity, was measured via the Total Histone H3 Acetylation Detection Fast Kit (Colorimetric, Abnova, Walnut, CA, USA) according to corresponding instructions.

### Statistical analysis

All experiments were performed more than 3 times. Statistical analysis was carried out using SPSS 16.0 (Chicago, IL, USA). The data in the present study were expressed as mean±SD and analyzed using two-way ANOVA followed by Turkey’s post hoc test or Kruskal-Wallis test followed by Dunn’s multiple comparisons test. *p* < 0.05 was significant statistically.

## Results

### Sirt1 was markedly decreased in CCI-induced neuropathic pain

**Figure 1.**
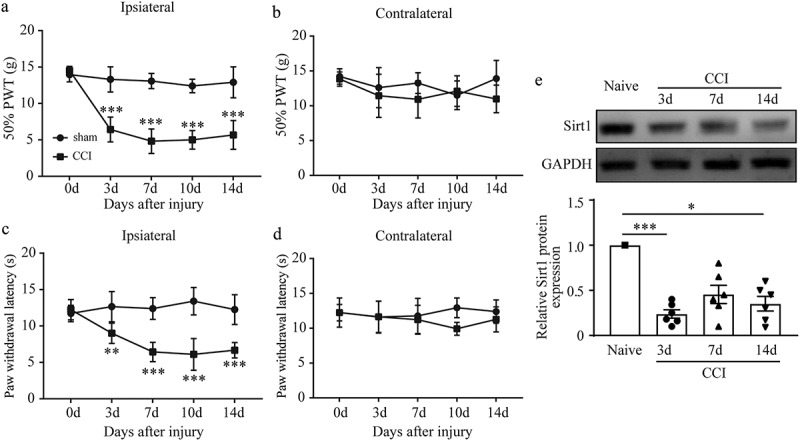
Sirt1 expression was significantly decreased in CCI–induced neuropathic pain model. (a-d) Ipsilateral and contralateral mechanical (a, c) and thermal (b, d) sensitivity in sham and CCI groups. (e) Sirt1 mRNA expression in L4-6 DRG in sham and CCI groups. (f) Sirt1 protein expression in L4-6 DRG in sham and CCI groups. *p < 0.05, **p < 0.01, ***p < 0.001 versus naïve group; repeated measured two-way ANOVA followed by Turkey’s post hoc test (a-d) or Kruskal-Wallis test followed by Dunn’s multiple comparisons test (e-f). n = 6 rats in each group.

In the present study, CCI surgery was performed to simulate neuropathic pain experimentally. Figure 1a and 1c showed that, the ipsilateral hind limb of CCI rats began to display lower PWT and PWL at and after day 3 after surgery, suggesting the symptoms of mechanical allodynia and thermal hyperalgesia. Also, no abnormality in behavioral tests was discovered in the contralateral hind limb of CCI rats (Figure 1b and 1d), indicating the successful construction of neuropathic pain model. To explore the relationship between Sirt1 and neuropathic pain, we first explored whether the expression of Sirt1 was changed in our established neuropathic pain model. As illustrated in Figure 1e and 1f, the mRNA and protein levels of Sirt1 in DRGs of CCI rats were significantly decreased compared with that in sham control group, hinting that Sirt1 might function in the development of neuropathic pain.

### Activating sirt1 alleviated CCI-induced pain hypersensitivity

**Figure 2.**
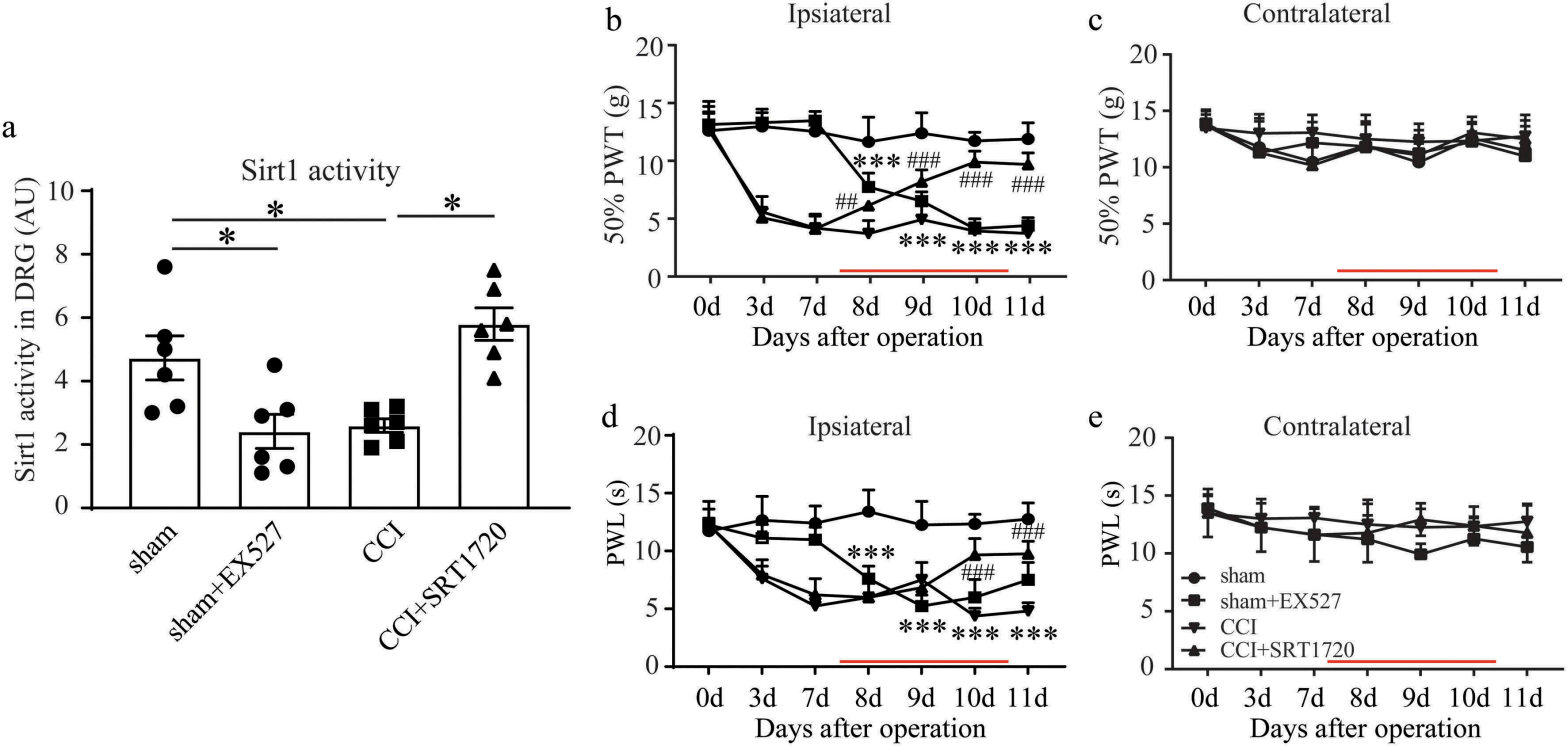
Activating Sirt1 alleviated CCI–induced pain hypersensitivity. (a) Sirt1 activity in L4-L6 DRG in sham, sham+EX527 (a Sirt1 inhibitor), CCI and CCI+SRT1720 (a Sirt1 activator) groups. (b-e) Ipsilateral and contralateral mechanical (b, c) and thermal (d, e) sensitivity; *p < 0.05, ***p < 0.001 sham+EX527 versus sham group. The time of drug injection is indicated as red lines. ^#^*p* < 0.05, *^##^p* < 0.01, ^###^*p* < 0.001 CCI+SRT1720 versus CCI group; by two-way (b-e) or one-way ANOVA followed by Turkey’s post hoc test (a). n = 6 in each group.

Next we explored whether the changes in Sirt activity could influence development of CCI pathology, EX527 (a known Sirt1 inhibitor) was administered to downregulate the Sirt1 activity in L4-L6 DRG. As shown in Figure 2a, Sirt1 activity was sharply decreased both in sham+EX527 and CCI groups. Results of behavioral tests illustrated that, rats in the above two groups exhibited decreased ipsilateral mechanical and thermal sensitivity (Figure 2b and 2d) 8 days after CCI operation, with no obvious effects on the contralateral hind limb (Figure 2c and 2e), indicating that Sirt1 inhibition could induce mechanical allodynia and thermal hyperalgesia which is similar with CCI operation. As the CCI rats showed higher Sirt1 activity, then a common Sirt1 activator, SRT1720, was administered to activate Sirt1 in CCI rats to explore whether Sirt1 could rectify or delay the neuropathic pain induced by CCI. Figure 2a showed SRT1720 indeed activated Sirt1 activity in CCI rats. Meanwhile Sirt1-activated CCI rats showed alleviated CCI–induced ipsilateral symptoms of neuropathic pain since 1 day after SRT1720 treatment (Figure 2b and 2d), suggesting the Sirt1 activation might be a useful intervening measurement for CCI–induced neuropathic pain.

### Resveratrol alleviated CCI-induced neuropathic pain partly through activating DRG Sirt1 in rats

**Figure 3.**
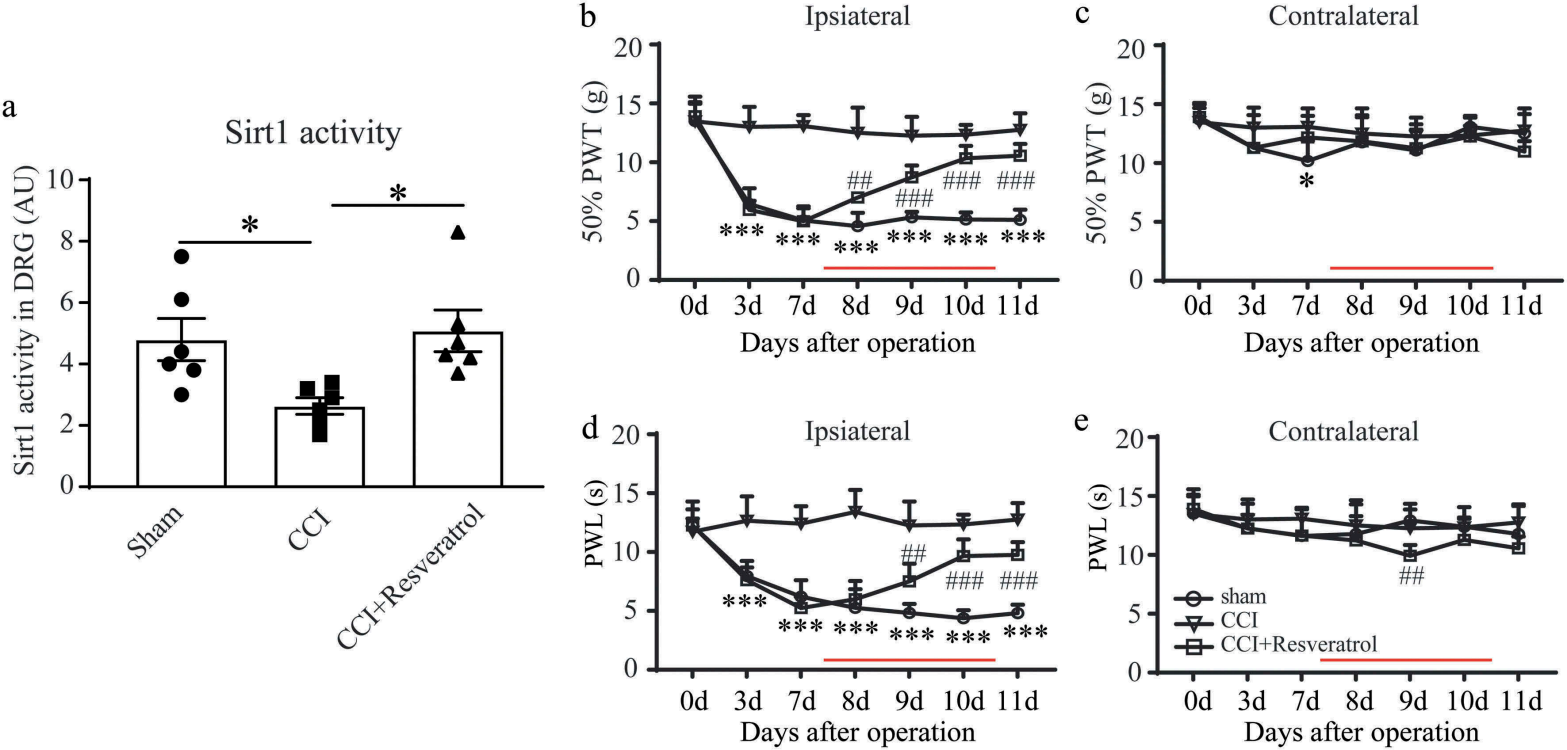
Resveratrol alleviated CCI–induced neuropathic pain through activating DRG Sirt1 in rats. (a) Sirt1 activity in L4-L6 DRG of sham, CCI, and CCI+Resveratrol group. (b-e) Ipsilateral and contralateral mechanical (b, c) and thermal (d, e) sensitivity in sham, CCI and CCI+Resveratrol groups. The time of drug injection is indicated as red lines. *p < 0.05, ***p < 0.001 versus sham group; *^##^p* < 0.01, ^###^*p* < 0.001 versus CCI group by one-way (a) or two-way (b-e) ANOVA followed by Turkey’s post hoc test. n = 6 in each group.

To develop novel and safe drugs in neuropathic pain therapy, resveratrol, the natural Sirt1 activator, was administered via intrathecal catheter 7 days after CCI surgery for 4 successive days. Sirt1 activity detection showed a significant increase in CCI rats treated with resveratrol (Figure 3a). Results of behavioral tests illustrated that, resveratrol treatment improved the ipsilateral symptoms of neuropathic pain of CCI rats from 8 days after operation, and almost didn’t influence the contralateral hind limb except a transient thermal hyperalgesia at day 9 (Figure 3b-3e). These results suggested that resveratrol might alleviate neuropathic pain induced by CCI surgery possibly through activating DRG Sirt1.

### Resveratrol increased miR-182 expression in CCI rats

**Figure 4.**
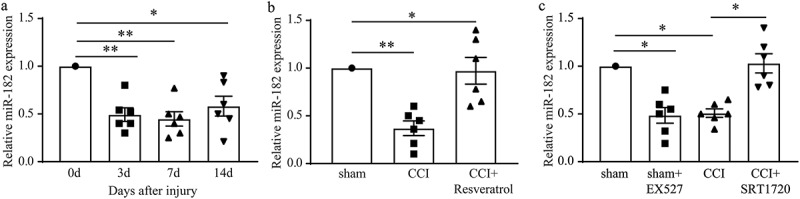
Resveratrol up-regulated miR-182 expression in CCI rats. (a) Time course of miR-182 expression in DRGs of CCI rats. (b) DRG miR-182 mRNA level in sham, CCI, and CCI+Resveratrol group at day 11 after surgery. (c) DRG miR-182 mRNA level in sham, sham+EX527, CCI and CCI+SRT1720 groups. *p < 0.05, **p < 0.01 by Kruskal-Wallis test followed by Dunn’s multiple comparisons test. n = 6 rats in each group.

The above results convinced us that Sirt1 possesses positive roles in the development of neuropathic pain. Next, we tried to explore the mechanism underlying this improvement of Sirt1 to neuropathic pain. miR-182 as the direct downstream effector of Sirt1 came into view. Figure 4a pointed that in CCI DRGs miR-182 was significantly increased during the development of neuropathic pain since 3 days after surgery. While resveratrol treatment reversed the low expression of miR-182 in CCI rats (Figure 4b), indicating that the activation of Sirt1 by resveratrol could increase the expression of miR-182 in the DRGs during the development of neuropathic pain. Additionally, Figure 4c shows that, Sirt1 inhibition by EX527 showed lowered miR-182 expression and Sirt1 activation by SRT1720 increased the down expression of miR-182 in CCI rats, illustrating Sirt1 expression could positively regulate DRG miR-182 expression.

### miR-182 mediated the resveratrol-alleviation of CCI-induced neuropathic pain

**Figure 5.**
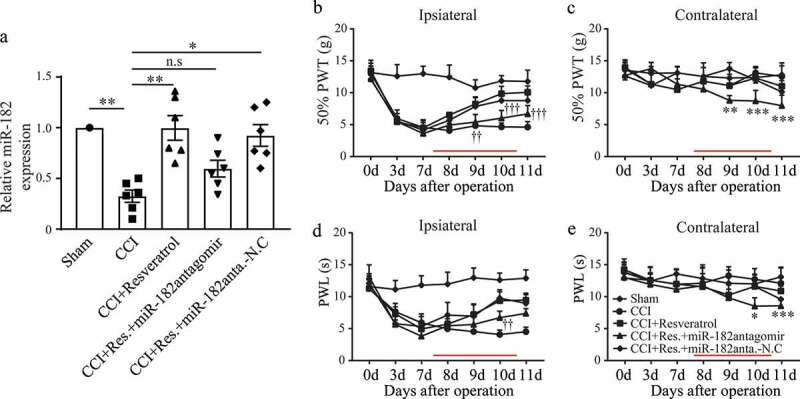
Alleviation of CCI–induced neuropathic pain by resveratrol was mediated by miR-182. (a) miR-182 expression level in L4-L6 DRGs. (b-e) Ipsilateral and contralateral mechanical (b, c) and thermal (d, e) sensitivity. The time of drug injection is indicated as red lines. *p < 0.05, **p < 0.01 versus sham group; ^†^p < 0.05, ^††^p < 0.05 and ^†††^p < 0.001 CCI+Res.+miR-182antagomir group versus CCI+Resveratrol group; Kruskal-Wallis test followed by Dunn’s multiple comparisons test (a) or two-way ANOVA followed by Turkey’s post hoc test (b-e). n = 6 rats in each group.

As it is clarified in the above mentioned that resveratrol upregulated the expression of miR-182 in CCI DRGs. In the following experiments, we planned to figure out the role of miR-182 in the analgesia of resveratrol to the neuropathic pain model. miR-182 antagomir was administered to inhibit the expression of miR-182 in resveratrol-treated CCI DRGs (Figure 5a). Figure 5b and 5d showed that, rats in CCI+Res.+miR-182 antagomir displayed lower 50% PWT and PWL of the ipsilateral hind limb, suggesting that miR-182 inhibition decreased the analgesia of resveratrol to neuropathic injury. Also, the contralateral hind limb in CCI+Res.+miR-182 antagomir group exhibited mechanical allodynia and thermal hyperalgesia (Figure 5c and 5e), which were absence in other groups, indicating that miR-182 inhibition not only alleviate the role of resveratrol but also affect contralateral hind limb sensation to pain stimuli. These results implied that resveratrol might alleviate CCI–induced neuropathic pain through upregulation of miR-182 in DRGs.

### Resveratrol down-regulated Nav1.7 expression in CCI rats

**Figure 6.**
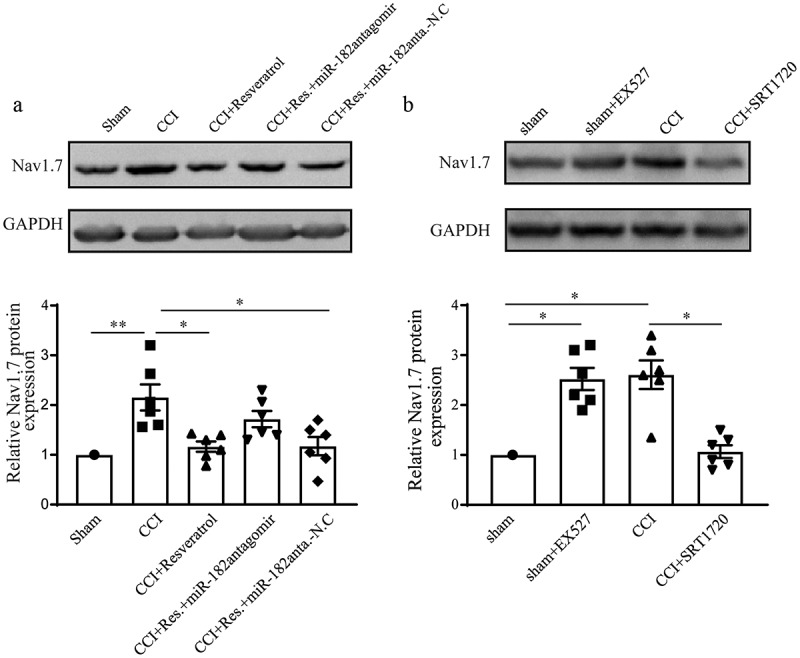
Resveratrol down-regulated Nav1.7 expression in CCI rats. (a) The expression of Nav1.7 protein in DRG from sham, CCI, CCI+Resveratrol, CCI+Res.+miR-182antagomir, and CCI+Res.+miR-182antagomir-N.C groups. (b) Expression of Nav1.7 protein in DRG from sham, sham+EX527, CCI and CCI+SRT1720 groups. *p < 0.05, **p < 0.01 versus sham group by Kruskal-Wallis test followed by Dunn’s multiple comparisons test. n = 6 rats in each group.

To further figure out the concrete mechanism underlying the alleviation of resveratrol to CCI–induced neuropathic pain, the expression of DRG Nav1.7 protein in different groups were detected via Western blot at day 11 after CCI. As shown in Figure 6a, CCI rats displayed highest Nav1.7 expression among all groups, while resveratrol treatment significantly decreased Nav1.7 expression, meaning that resveratrol might alleviate neuropathic pain through downregulation of Nav1.7. Rats in CCI+Res.+miR-182antagomir group displayed higher Nav1.7 expression than CCI+Resveratrol group, that is, miR-82 inhibition increased Nav1.7 expression which was downregulated by resveratrol, indicating that resveratrol might downregulate Nav1.7 expression via upregulating miR-182 expression. Also, we tested the direct influence of Sirt1 activity on Nav1.7 protein expression, and found that Sirt1 inhibition upregulated Nav1.7 expression and Sirt1 activation significantly downregulated its expression (Figure 6b), suggesting that Sirt1 negatively affects Nav1.7 expression.

## Discussion

The present study aimed to explore the effect of resveratrol on CCI–induced neuropathic pain and figure out the downstream target effectors involved in this process. We constructed neuropathic pain rat via CCI surgery, evidenced by mechanical allodynia and thermal hyperalgesia. The CCI rats displayed lower Sirt1 and miR-182 expression as well as higher Nav1.7 expression, which would be almost reversed by resveratrol treatment for 4 successive days. We also found that miR-182 expression inhibition erased the analgesia effect of resveratrol to CCI–induced neuropathic pain possibly through upregulating Nav1.7 expression. This study explored for the first time the relationship between resveratrol-activated Sirt1 and the miR-182/Nav1.7 downstream target in the development of CCI–induced neuropathic pain.

Sirt1 is a nicotinamide adenine dinucleotide (NAD)-dependent protein lysine deacetylase and is well acknowledged as a mediator of longevity in yeast [19]. Many proteins are substrates of Sirt1 deacetylation, including histones, and non-histones, such as p53, FOXO1, NF-κB, PGC1α, and more [20]. Sirt1 reduces pro-apoptotic effect of p53, suppresses the pro-inflammatory effect of NF-κB, while activates PGC-1α to increase glucose levels, mitochondrial biogenesis, and insulin sensitivity. These above effects, combined with other ones, contribute to the longevity effect of Sirt1 [17]. Sirt1 is predominantly expressed in neurons and has been reported to be involved in neurological disorders, including neuropathic pain. SIRT1 suppresses β-amyloid production by activating the α-secretase gene ADAM10 and induces Notch signaling pathway, illustrating its potential in the treatment of Alzheimer’s disease [21]. The spinal Sirt1 deacetylase activity decreased early and significantly along the development of CCI–induced neuropathic pain, and pharmaceutical activation of Sirt1 exhibited transient analgesia effect [22]. The enhancement of Sirt1 activity may be a promising strategy for the prevention and treatment of neuropathic pain and neurological disorders. As the agonist of Sirt1, resveratrol downregulates the Sirt1 protein expression levels and protects neurons from stroke via the Sirt1-dependent manner to elicit ischemic tolerance in the cerebral ischemia condition [23]. Furthermore, resveratrol has been shown to relieve several types of neuropathic pain via targeting Sirt1 or other pathways. It is reported that resveratrol perform through enhancing IL-4 receptor-mediated anti–inflammatory effects in the spinal cord and relieves neuropathic pain induced by sciatic nerve injury [24]. Several studies focus on the alleviation of resveratrol-activated Sirt1 to neuropathic pain induced by chemotherapeutic drugs, and nerve ligation injury, while the concrete mechanisms remains to be further illustrated [5,6,22,25].

MicroRNAs (miRNAs) are the small noncoding RNA that is found in multicellular eukaryotes and have fundamental roles in the regulation of genes related with important biologic processes. miR-182 is identified as the downstream effector of Sirt1, and its expression is upregulated by Sirt1 overexpression in trigeminal neurons, promoting diabetic corneal nerve regeneration [26]. Also, miR-182 is reported to relieve neuropathic pain induced by spared nerve injury [14]. While the relationship between Sirt1 and miR-182 hasn’t been elucidated in neuropathic pain yet.

The pain signals are transmitted along the DRGs to the spinal cord, finally to the corresponding zone of the brain. Hence as the soma assembly of the first-order neurons for the pain pathway, the excitability of DRGs is crucial for the pain signaling, which is mostly dependent on the ion channels. Nav1.7 belongs to the voltage-gated sodium channels and is mainly expressed in DRGs and sympathetic ganglia. Ahn HS et al. reported that mutant Nav1.7 depolarizes the resting membrane potential to induce an enhanced inward current, and contributes to hyperexcitability of DRG neurons [27]. Evidences showed that DRG Nav1.7 was associated with pain-related disorders. For instance, Nav1.7 mutant reduced the threshold of single-action triggering potential, meaning that it induced DRG hyperexcitability which contributes to inherited erythromelalgia/erythermalgia [28]. Also Nav1.7 is illustrated to play important roles in the development of neuropathic pain. Nav1.7 protein levels were notably increased in L4-L5 DRGs under CCI conditions and is suggested to be one promising effector protein correlated with pathogenesis of neuropathic pain[13]. Considering that miR-182 is the downstream effector of Sirt1 and miR-182 downregulates Nav1.7 expression to relive neuropathic pain in a previous study [14], we hypothesized that Sirt1 might downregulated Nav1.7 expression via miR-182. And our findings further confirmed this hypothesis. Yet little is known about the concrete mechanism between miR-182 and Nav1.7, the studies about microRNAs and Nav1.7 might supply some clues. lncRNA X inactivate-specific transcript (XIST) suppression is reported to act as a sponge of miR-146a, suppress the activation of DRG satellite glial cells, and reduce secretion of inflammatory cytokines and result in the alleviation of inflammatory pain [29]. Further study needs to be performed concerning this aspect in the future.

In the present study, there were several limitations. On one hand, Sirt1 inhibition should be performed to reversely confirm the conclusion that resveratrol conducts analgesia effect through activating Sirt1 in the DRGs and Sirt1 directly regulates miR-182. On the other hand, we only detected the expression of Nav1.7 after resveratrol treatment with or without miR-182 inhibition. Whether the abnormal expression of Nav1.7 could result in further changes in depolarization, inward currents and excitability of DRG neurons would be explored in the future. Meanwhile the present study hasn’t come to the relationship between Sirt1 activity and Nav1.7 expression, which will be investigated in the following studies.

In summary, resveratrol alleviated CCI–induced neuropathic pain, possibly through activating Sirt1 and upregulating miR-182 expression in CCI DRGs to suppress Nav1.7 expression, to a large extent.
